# Root and Canal Morphology of Mandibular Second Molar in an Iranian Population by Clearing Method

**Published:** 2013-06

**Authors:** M Zare Jahromi, F Jafari Golestan, M Mashhadi Esmaeil, SH MoouaviZahed, M Sarami

**Affiliations:** a Dept. of Dentistry, Khorasgan Branch, Islamic Azad University, Isfahan, Iran.; b Dept. of Endodontics, School of Dentistry, Islamic Azad University, Khorasgan Branch, Isfahan, Iran.; c Dentist

**Keywords:** Canal Morphology, Mandibular Second Molar, Clearing, Iranian Population

## Abstract

**Statement of Problem:** The knowledge of the pulp anatomy plays an important role in the success of endodontic treatments.

**Purpose:** The aim of this study was to determine the root and canal morphology of the mandibular second molar teeth in an Iranian population.

**Material and Methods: **One hundred intact human mandibular second molars were collected. The teeth were examined visually and the number of their roots were recorded. The teeth were covered using of lacquer. Access cavities were prepared and the pulp tissue was dissolved by sodium hypochlorite. The apices were covered with the glue and the root canals were injected with the methylene blue and were decalcified with 10% nitric acid, dehydrated with ascending concentrations of alcohol and rendered clear by immersion in methyl salicylate. The following remarks were evaluated: (i) number of root canals per tooth; (ii) number of canals per root; (iii) canal configuration in each root.

**Results: **Of 100 examined teeth; 6% had one root, 89% had two roots, 2% had three roots and 3% had C-shaped roots. The teeth were classified based on the number of canals: 3 % had single canal, 6 % two canals, 54% three canals, 34% four canals, whilst 3 % had C-shaped roots. Based on the Vertucci classification, the most prevalent canal configuration in the mesial root was type II and in the distal root was type I.

**Conclusion** ***:*** Iranian mandibular second molar teeth exhibit features which are similar to the average Jordanian, Caucasian and Burmese root and canal morphology.

## Introduction

One of the reasons of the failure in the dental root canal treatment is the lack of enough knowledge of the pulp anatomy and canal variation. The type of the normal anatomy of the pulp and the possible different changes in its anatomy should be known. In addition of knowing the different types of normal and abnormal pulp; a dentist should be able to use the special techniques to choose the type of anatomy inside the pulp during the treatment [1-2]. It is crucial to know some important issues in the anatomy of the root canals such as the root numbers, number of canals in each root and their position, cross-section of root in term of length and width, the most prevalent curvature (especially in the buccoligual sections)and outline form of each root in all dimensions [3-5]. In a study done by Ingle et al. [3], on mesial and distal root of the second molar of the lower jaw, the following results were given: in the mesial root 13% type I, 49% type II, 38% type III and in the distal root 92% type I, 5% type II, 3% type III canals were reported. In the study done by NilaKantan et al. [6] on the morphology of the canal and the root of the second mandibular molar, it was shown that a lot of teeth (87.8%) had two separated root with three canals. The morphology of the c-shaped canal in 7.5% of the teeth under study was seen. Both the mesial and the distal root of these teeth showed different types of shape and numbers of the canals with the most prevalence of type I and IV [6]. In the study of De Pablo et al. [7]; the prevalence of third root of the second molar was reported about %13 and there was a strong relation with this finding and the race of the people whose teeth were examined in the study. Three canalled were seen almost in %61.3, four canalled in %35.7 and five canaled in %1 of the samples. The mesial root was seen in %94.3 of two canaled teeth and in %2.3 of three canalled teeth. The most prevalent type of root canal system was type IV of Vertucci (%52.3) and then type two (%35)0.The type of root canal in distal root was %62.7 in type I, %14.5 in type II and %12.4 in type IV [7]. Sadeghi et al. [8] studied on the first and second mandibular molar teeth in an Iranian population and reported that the prevalence of different type of canals in mesial root of second mandibular molars were as follows: 6% type I, 26% type II, 62% type IV, 4% type V, 2% type VI and in 10% accessory canals and in 8% lateral canals were seen, in distal root were 88% type I, 6% type II, 6% type IV and in 10% accessory canals, in 4% lateral canals and in 28% accessory canals in bifurcation were seen [8]. In a research done by Al-Qudah and Awawdeh in 2009 [9] who studied the canal and the root morphology of first and second mandibular molars, the second molar teeth were three- canalled in %58, two- canalled in %19 and four- canalled in %17 and C-shaped canal in %10 . The most prevalent type of root canals in the mesial root of second molar (%40) was type IV of Verttuci system and in the distal root (%79) was type I [9]. Maning et al. showed that of the 149 studied teeth, 22 per cent had single roots, 76 per cent had two roots and 2 per cent had three roots [10]. In another study he showed that The C-shaped canals were found more frequently in Asians than in other races [11]. Ashraf et al. in 2003 [12] showed that 13.8% of the second molar which were studied from an Iranian population had C-shaped canals. In a study of Rahimi et al. [13] ; carried out in 2008 on the first and second mandibular molars in an Iranian population, showed that 86.3% of mandibular second molars had two roots, 9.3% had one root and 4.3% had three roots. Ninety percent of the mesial roots of the mandibular second molars with double roots had two canals (predominantly with a type II or III configuration) and 77.5% of the distal roots of mandibular second had one canal (predominantly with a type I configuration). Among the mandibular second molars, 7.2% had C-shaped canals and these configurations were seen mostly in the single-rooted mandibular second molars.In a study conducted by Sachdeva et al. on the second mandibular premolar using spiral tomography showed that the deviation in the canal anatomy occurs naturally. 

Basic knowledge of the canal anatomy and its variation for successful root canal treatment is necessary [14]. In the study enrolled by Gleghorn et al. [14] which compared the first and the second mandibular premolars; have showed that genetic and racial variations may cause differences in the number of roots and canals in the human population. Most teeth with accessory canals and roots were reported in Chinese, Australian and African populations [14-15]. However, these studies were mainly performed on North American, Jordanian, Caucasian, Turkish and Chinese populations. There are no published reports on the root canal anatomy of the mandibular second molars in the Iranian population. The aim of this study was to investigate the root canal anatomy of the mandibular second molars in an Iranian population using Vertucci classification and to compare these findings with the published reports of different population.

## Material and Methods

One hundred extracted human adult mandibular second molar teeth from an Iranian population (]sfahan City) were collected by three endodontists. Teeth with fracture, incompletely formed roots, metallic restorations, and deep caries were not included. Calculus and stains were removed by using an ultrasonic scaler. They were radiographed by using a digital radiography set from 3 buccal, mesial and distal angles and were encoded. Access cavities were prepared using No. 2 round bur (Tizkavan; Tehran, Iran), the orifice were checked by an endodontic explorer and the pulp tissue was dissolved by using 2.5% sodium hypochlorite (Tage; Iran) for 12 hours. The teeth were then rinsed under running tap water for two hours and dried overnight. After drying, except for the apex region, other parts of the teeth were covered by two layers of lacquer (Lilium; Iran) and the apices were covered with liquid glue (Razi; Iran). To stain the samples, a syringe with a gauge 27 needle was used to inject the 2% methylene blue solution (Merck; Germany) from the crown into the root canal spaces. The teeth were then air-dried and decalcified in 5% nitric acid (Merck; Germany) in 37ºC for 4 to 5 days. The acid solution was changed daily and the finishing point of decalcification was determined by successive radiographs. The teeth were washed under running water to remove the traces of nitric acid, dried and dehydrated using ethanol (70%) (Merck; Germany) for 24 hours and then with ethanol (95%and 100%) for one hour; respectively. Finally the teeth were rendered transparent by immersing in methyl salicylate (Merck; Germany). The cleared teeth were examined under stereomicroscope with 7.5X magnification (MJC IO; Moscow, Russia). The canal configurations were categorized into the first seven types of Vertucci classification (1984) as follows:

1. Type I. A single canal present from the pulp chamber to the apex;
2. Type II. Two separate canals leave the pulp chamber and join near the apex to form one canal;
3. Type III. One canal leaves the pulp chamber, divides into two canals within the root, and then merges to exit in one canal;
4. Type IV. Two separate and distinct canals are present from the pulp chamber to the apex;
5. Type V. Single canal leaves the pulp chamber but divides into two separate canals with two separate apical foramina;
6. Type VI. Two separate canals leave the pulp chamber but join at the midpoint and divides again into two separate canals with two separate apical foramina; and
7. Type VII. One canal leaves the pulp chamber, divides and rejoins within the canal and finally re-divides into two distinct canals near the apex.

## Results

A total of 100 studied mandibular second molar teeth were initially classified based on their root number, in which 6% had one root, 89%had two roots, 2 % had three roots and 3% were C-shaped teeth. Then the teeth were classified based on the number of canals. There were 6 % two-canals, 54% three-canals, 34% four-canals, 3% single-canal and 3% C-shaped teeth. The classification of the canals in a root was done based on the Verttuci classification. All one- canalled teeth (no=3) were classified as type I. In the group of two- canalled teeth (No=6), three (%50) were two rooted in which all (%100) had one canal in each root, three (%50) were one rooted and all (%100) were type II. In the group of three- canalled teeth (No=54) that were two rooted; all (%100) had one canal in the distal root. Both the mesial and distal roots of the two rooted molars showed variations in the canal number and configuration. Out of the 34 teeth classified in the four canalled group; 32(94%) were two rooted and two (6%) were three rooted. In the group of the two rooted teeth, in mesial roots, 6(9%) were type II, 25(78%) were type III and one was (3%) type IV, and in distal root 11(35%) were type II, 9(28%) were type III, 12(37%) were type IV. Type I, type II and type III canal anatomies were most common in the mesial and the distal roots of the two- rooted second molars, respectively. In the group with three- rooted teeth, in mesial roots, all (%100) were type II and in distal roots, all (%100) were type I.C-shaped canal morphology was observed in 3% of the studied teeth.

## Discussion

One of the predominant causes of the failure of root canal treatment in mandibular second molar is the variations in root canal anatomy [2]. This study examined the root canal morphology of the mandibular second molar teeth in an Iranian population. Many studies have been carried out on the root canal anatomy using different methods such as: macroscopic section, radiography, direct observation with microscope, decalcification and clearing, 3D reconstruction and computed tomography. Among all these methods; decalcification and clearing technique has provided the most detailed information along with being simple and inexpensive [5-7]. Canal negotiation with instruments is unneeded in this technique, thereby the original form and relation of the canals are maintained and a three-dimensional view of the root canal is provided. Of the 100 teeth, 6% had single roots in the current study and it was 9.3% in Rahimi et al. study [14] but in Maning et al. study, it was 22% [6, 13]. In the current study; 2% of the teeth had three roots which is similar to the findings of Rahimi et al. [3], Maning et al. and Al-Qudah & Awawdeh studies [9-10, 13]. In this study, 3% of the teeth were C-shaped but in the Neelakantan et al. study [6] this percent was 7.5%, in Gulabivala et al. study [16] this percent was 22.4%, in Rahimi et al. study [13] was 7.2%, Ashraf et al. found 13.8% and Al-Qudah & Awawdeh et al. found 10% [6, 9, 12-13,16]. There was a high prevalence of two-rooted mandibular second molars. A total of 89% of the mandibular second molar teeth were found to have two roots which is similar to the findings of Maning et al. (76%)[10], Neelakantan et al. [6] (87.8%) but in Gulabivala  et al. study [16] this percent was 58% .The majority of teeth had three (54%) canals and this result is similar to the study of Gulabivala et al. [16]. The most prevalent canal pattern in this study was type III (78%) in the mesial root of the mandibular second molar followed by type II and type IV which is similar to the findings of Sadeghi et al., Al-Qudah & Awawdeh and Gulabivala et al. [9, 16]. The most prevalent canal pattern in distal root was type I which is similar to the finding of Neelakantan et al. [6], Al-Qudah et al. [9], Gulabivala et al. [6] [6, 8-9, 16]. Iranian mandibular second molar teeth exhibit the features similar to the average Jordanian, Caucasian and Burmese root and canal morphology.

## Conclusion

An accurate knowledge of the morphology of the pulp cavity is rationally crucial before any endodontic procedure. Radiographs, exposed at two different horizontal angles and their careful interpretation, would facilitate finding the additional root canals. There was a prevalence of three-rooted and C-shaped roots (or canals) in mandibular second molars in an Iranian population. Conical roots tend to have simple canal systems, whilst wider roots have more complex canal systems.
